# Prevalence of anxiety symptoms in a Ugandan population sample and psychometric properties of the Generalized Anxiety Disorder-7 scale (GAD-7) in Luganda and Runyoro

**DOI:** 10.1186/s12888-025-06944-8

**Published:** 2025-05-19

**Authors:** Leo Ziegel, Cristina Espinosa da Silva, Robert Bulamba, Alex Daama, Grace Kigozi, Amanda P. Miller, Godfrey Kigozi, Emmanuel Kyasanku, Stephen Mugamba, Anders Hammarberg, Anna Mia Ekström, Fred Nalugoda, Anna-Clara Hollander

**Affiliations:** 1 Department of Global Public Health, Karolinska Institutet, Solna, Stockholm, 17177 Sweden; 2 Department of Medicine, University of California San Francisco, San Francisco, CA USA; 3 Africa Medical and Behavioural Sciences Organization, Nansana, Uganda; 4 School of Public Health, San Diego State University, San Diego, CA USA; 5 Centre for Psychiatry Research, Department of Clinical Neuroscience, Karolinska Institutet and Stockholm Health Care Services, Region Stockholm, Stockholm, Sweden; 6 Department of Infectious Diseases (Venhälsan), South General Hospital, Stockholm, Sweden

**Keywords:** Generalized Anxiety Disorder-7, GAD-7, Anxiety symptoms, Anxiety disorders, Population study, Uganda, Sub-Saharan Africa

## Abstract

**Background:**

Locally validated measures are required for robust clinical and epidemiological assessments of anxiety symptoms and disorders. Few studies on the African continent have examined the psychometric properties of the Generalized Anxiety Disorder-7 scale (GAD-7). We aimed to investigate certain psychometric properties of GAD-7 translated into Runyoro and Luganda, two Ugandan languages, and to measure the prevalence of anxiety symptoms in a Ugandan population sample.

**Methods:**

Data were collected in 2021–22 through the African Medical and Behavioural Sciences Organization (AMBSO) Population Health Surveillance (APHS), a population cohort study in Uganda. GAD-7, Patient Health Questionnaire-9 (PHQ-9), and questions on socio-demographic factors were administered during individual face-to-face interviews. Construct validity, internal consistency, and concurrent validity of the Runyoro- and Luganda-translated GAD-7 were examined using confirmatory factor analysis (CFA), two internal consistency coefficients (Revelle’s omega total and Cronbach’s alpha), and Pearson’s correlation coefficient, respectively. The prevalence of anxiety symptoms and likely anxiety disorders was also estimated.

**Results:**

A total of 4107 individuals aged 13–80 participated, with 2206 and 1901 speaking Runyoro and Luganda, respectively. The Runyoro-translated GAD-7 retained its one-factor structure (RMSEA = 0.097, CFI = 0.977, TLI = 0.966), had good internal consistency (omega total = 0.85), and correlated moderately with the PHQ-9 (*r* = 0.67, *p* < 0.01). The Luganda-translated GAD-7 also fit a one-factor structure (RMSEA = 0.097, CFI = 0.989, TLI = 0.983), exhibited excellent internal consistency (omega total = 0.90), and strong concurrent validity with PHQ-9 (*r* = 0.71, *p* < 0.01). Overall, participants reported low levels of anxiety symptoms. Using GAD-7 ≥ 10 binary cut-offs, the total prevalence of likely anxiety disorders was 1.5% (0.5% among males, 2.2% among females).

**Conclusions:**

GAD-7 was translated into Runyoro and Luganda, two Ugandan languages, and both translations showed good psychometric properties. The prevalence of likely anxiety disorders was low in this diverse large Ugandan population sample, the largest population study on anxiety in Uganda to date. The low prevalence could partly be due to individuals with more severe anxiety symptoms not participating or GAD-7 not including local idioms of anxiety. Further clinical validation is required.

**Supplementary Information:**

The online version contains supplementary material available at 10.1186/s12888-025-06944-8.

## Background

Mental disorders constitute a substantial public health burden globally. Anxiety disorders are estimated to be the most prevalent mental disorders, but estimates differ widely between cultures and countries [1]. Examples of anxiety disorders include generalized anxiety disorder (GAD), different phobias and panic disorder, previously also post-traumatic stress disorder (PTSD) and obsessive–compulsive disorder (OCD). Effective therapeutic and pharmacological treatments for anxiety disorders exist, but access is insufficient in many countries due to inadequate funding for prevention, detection, or treatment of mental disorders in general [2, 3].

Validated measures are needed to accurately assess the burden of disease attributable to anxiety disorders and inform allocation of resources aimed at addressing this public health issue. The Generalized Anxiety Disorder-7 scale (GAD-7) and Patient Health Questionnaire-9 (PHQ-9) were jointly developed in the early 2000s to screen US-American primary care patients for GAD [4] and major depressive disorder [5] because of the high prevalence and overlap of these disorders. GAD-7 has since then been widely adopted for research purposes [6] and is freely available in multiple languages [7]. All cultures have local idioms of distress, i.e. common expressions to describe different types of suffering, and local idioms are important to consider when translating constructs such as anxiety [8].

Any screening tool to be used outside of its original setting requires both psychometric and clinical validation to ensure local utility and reliable estimates [9, 10]. Psychometric validation evaluates a scale’s structure and quantitative properties (e.g., construct validity, internal consistency, and concurrent validity) while clinical validation compares a screening tool with a gold standard, which for mental disorders usually consists of a structured interview or assessment by trained professionals. Clinical validation occurs less frequently, in part because it requires trained clinical providers to administer the diagnostic assessment. Although GAD-7 has been used previously in Uganda [11, 12], is has not been validated psychometrically or clinically. Previous assessments from other low and middle-income countries have found GAD-7 to be psychometrically robust across diverse settings, such as in Malaysia, Peru, and Kenya [13–15].

As evidenced in a recent meta-analysis of the PHQ-9 [16], epidemiological studies using screening tools to measure the burden of mental disorders frequently overestimate their true prevalence. However, screening tools are easier to administer, faster and less expensive than structured interviews and, when carefully interpreted, can provide valuable information for mapping and comparing mental illness globally.

In a 2020 systematic review, GAD-7 and Kessler-10 were found to be the most validated anxiety screening tools in low and middle-income countries, with wide ranges of sensitivities and specificities [17]. In a 2016 global meta-analysis, GAD-7 had an 83% pooled sensitivity and 84% pooled specificity for identifying GAD using the cutoff ≥ 8 points. Accordingly, GAD-7 can yield high numbers of false positive and false negative responses in settings with low prevalences (like in population surveys), and the actual prevalence of anxiety disorders is usually substantially lower than screening results [18].

A 2021 scoping review of GAD in Africa reported broad heterogeneity in point prevalence estimates (0.7–9.6%, with one outlier at 36.5%) in general population samples and cautioned against the use of screening tools (such as GAD-7) in assessing prevalence [19]. A 2021 systematic review of studies among Ugandan adults found a higher pooled prevalence of anxiety disorders (20%) than regional estimates. However, it is worth noting that these estimates included GAD as well as PTSD, a condition disproportionately burdening Ugandans residing in historically war-affected regions [20].

In this study, we aimed to investigate certain psychometric properties of the Runyoro- and Luganda-translated GAD-7, and to measure the prevalence of anxiety symptoms in a Ugandan population sample using the psychometrically validated measures.

## Methods

### Setting

This study used data from the Africa Medical and Behavioural Sciences Organization (AMBSO) Population Health Surveillance (APHS), an ongoing population-based cohort study in Uganda. Methods of the APHS have been described in detail previously [21]. APHS is conducted annually across six communities (one urban, semi-urban and rural per district) in Hoima and Wakiso, two districts in mid-Western and central Uganda, respectively. Varying community types were selected to represent different characteristics of the Ugandan society, including population density, availability of public infrastructure and income-generating activities. The main language in Hoima is Runyoro. The predominant language in Wakiso is Luganda, which is also the most widely spoken language in the country.

### Data collection

Data were collected during the third round of APHS from May 2021 to July 2022. Each round of data collection consists of census activities to enumerate the population and collect household sociodemographic information, followed by individual face-to-face survey administration and collection of biological samples at a community hub. All censused individuals aged 13 years and older were invited to participate in the survey. Surveys lasted approximately one hour, and participants were compensated for their time and any transport costs. Prior to participation, written informed consent (or assent for minors) was obtained. APHS was approved by the local Clarke International University-Research Ethics Committee (CIUREC/0059) and registered with the Uganda National Council for Science and Technology (UNCST/SS4468).

### Survey translation

The survey was developed in English, then translated into Runyoro and Luganda by trained social workers fluent in each language and designated by the Common European Framework (CEFR) as C1 or C2 proficient English speakers. Translations were checked by senior researchers to be accurate and locally meaningful and then pilot tested with staff who were not involved in the translation process. Table 1 presents the Runyoro and Luganda versions of GAD-7.

**Table 1:** GAD-7 in the original English version and translated into Runyoro and Luganda

	**Original**^**a**^	**Runyoro**^**b**^	**Luganda**^**c**^
**Prompt**	Over the last two weeks, how often have you been bothered by the following problems?	Mu wiki 2 ezihingwire, mirundi eingaha eyotalibanizibwemu obuzibu bunu hansi?	Mu wiki 2 eziyise, mirundi gyenkanawa gyotaataganyiziddwako obuzibu buno wamanga?
**GAD1**	Feeling nervous, anxious, or on edge	Okuhuura otiinire	Okuwulira nga otidde
**GAD2**	Not being able to stop or control worrying	Obutasoboora kulekeraho kweralikirira	Obutasobola kulekeerawo kweralikirira
**GAD3**	Worrying too much about different things	Okweralikira okuhinguraine habintu ebikwahukana	Okweralikiria ekisusse ku bintu eby’enjawulo
**GAD4**	Trouble relaxing	Okusanga obuzibu mukwehumuzamu	Okusanga obuzibu okwewumuzaamu
**GAD5**	Being so restless that it is hard to sit still	Obutatekana mukichweka	Obutatereera mu kifo
**GAD6**	Becoming easily annoyed or irritable	Okwebihizabihiza	Okwenyiizanyiiza
**GAD7**	Feeling afraid, as if something awful might happen	Okuhulira owekangire ngu ekintu ekibi kisobora kubaho(kubikirwa)	Okuwulira nga otidde/Okufuna ekyebikkiro

^a^Response options in English: 0 = not at all, 1 = several days, 2 = more than half the days, 3 = nearly every day. No permission required to reproduce, translate, display or distribute [6]

^b^Response options in Runyoro: 0 = tekibaireho nakatoito, 1 = biro bitaito, 2 = biro bingi, 3 = haihi buli kiro

^c^Response options in Luganda: 0 = tekibaddeewo nakatono, 1 = naku ntono, 2 = enaku nyingiko, 3 = kumpi buli lunaku

### Measurements

*Anxiety symptoms* in the last two weeks were measured using the 7-item GAD-7 with 4-point Likert-type responses: 0 = not at all, 1 = several days, 2 = more than half of the days, 3 = nearly every day (Table 1). The GAD-7 scale follows the Diagnostic and Statistical Manual of Mental Disorders (DSM)-IV-TR criteria for GAD. A summed score was created from the seven scale items, with higher scores indicating more severe anxiety symptoms (range 0–21). Additionally, continuous GAD-7 scores were categorized using the original 4-category cut-offs (0–4 = no or minimal anxiety, 5–9 = mild anxiety, 10–14 = moderate anxiety, 15–21 = severe anxiety), and dichotomized (0–9 = anxiety disorder unlikely, 10–21 = likely anxiety disorder) for comparability with other published literature [4].

*Sociodemographic factors* included age, sex, highest education level, household socioeconomic status, and community type. Age was self-reported in completed years. Sex (male or female) was determined by interviewer observation. Highest education level was categorized as none, primary (P1-P7), secondary (S1-S6), and tertiary education. Household socioeconomic status was assessed with an asset index using standard methodology from the Demographic and Health Surveillance Program [22], with quartiles calculated at the household (not individual) level. Urbanity status of communities (i.e., urban, semi-urban, or rural) was defined at cohort creation according to inter alia density of households, the existence of shops, places for social gatherings, employment opportunities, and means of transportation.

*Depressive symptoms* were measured using the 9-item Patient Health Questionnaire (PHQ-9) [5], which was previously translated and assessed psychometrically by the study team in the same cohort [23]. PHQ-9 measured depressive symptoms in the past two weeks using 4-point Linkert-type responses (0 = not at all, 1 = several days, 2 = more than half the days, 3 = nearly every day). A summed score was created from the nine item responses, with higher scores indicating greater depressive symptomology (range: 0 – 27).

### Statistical analysis

Of the individuals who participated in the third round of APHS (*N* = 4108), the present analysis was restricted to those with complete GAD-7 data (*N* = 4107). Descriptive statistics were estimated to characterize the study population overall as well as by language (i.e., Runyoro or Luganda). Means (with standard deviations [SD]) and medians (with interquartile range [IQR]) were used for continuous variables and frequencies and percentages were used for categorical variables. Psychometric properties assessed in each language included the translated scale’s construct validity, internal consistency, and concurrent validity.

*Construct validity* was evaluated using confirmatory factor analysis (CFA) to determine whether the structure of the scale in the current sample corresponded to its designed one-factor structure, whereby all seven scale items share one common underlying characteristic. Factor loadings were calculated using the *cfa* function with a diagonally weighted least squares estimator in the *lavaan* package in R [24] and estimated for each scale item the proportion of the variance that was explained by the underlying factor. The more variance accounted for, the stronger the agreement with the one-factor structure [25]. Model fit was evaluated using three model fit indices: the Root Mean Square Error of Approximation (RMSEA), Comparative Fit Index (CFI), and Tucker-Lewis Index (TLI). RMSEA values below around 0.1 indicate acceptable model fit [26], CFI and TLI values above around 0.9 indicate good model fit.

*Internal consistency* is a type of reliability that measures the homogeneity of the items that comprise a scale. More specifically, reliability is high when the proportion of variance attributable to random error is small compared to a scale score’s total variance. Internal consistency is not a feature of the scale itself but varies based on study population and setting. Longer scales and more heterogeneous samples have a larger total variance and, subsequently, have higher internal consistency [27, 28]. Two reliability coefficients (i.e., Revelle’s omega total and Cronbach’s alpha) were used to assess the internal consistency of the translated GAD-7 scales using the *omega* function in the *psych* package in R [29]. Revelle’s omega total is a more robust measure of reliability due to its fewer and more realistic underlying assumptions [28], while Cronbach’s alpha coefficient was estimated for comparison with existing literature.

*Concurrent validity* assesses how well a measure correlates with similar or related measures at the same point of measurement [30]. A Pearson’s correlation coefficient between anxiety symptoms (GAD-7 score) and depressive symptoms (PHQ-9 score) was estimated using the *pwcorr* statement in STATA. Correlations were classified as weak (*r* < 0.3), moderate (*r* = 0.4–0.6), or strong (*r* > 0.7) [31].

The prevalences of anxiety symptoms and likely anxiety disorders were estimated, with the latter stratified by sex for comparability with other published literature. Due to small sample sizes, no further subgroup analyses by sex were performed.

Analyses were performed in STATA SE Version 16.1 and R Studio Version 3.6.1.

## Results

Overall, participants had a median age of 29 years (IQR 21–39 years), 2444 (59.5%) were female, 2056 (50.1%) had at least some secondary education, and community types were evenly distributed within districts as designed (Table 2). Age, sex, and education level distributions were approximately comparable between Runyoro-speaking participants in Hoima and Luganda-speaking participants in Wakiso. Compared to Luganda-speaking participants in Wakiso, a greater proportion of Runyoro-speaking participants in Hoima had higher socioeconomic status (70.1% in the upper two quartiles compared to 42.6%, respectively) and fewer lived in a semi-urban community (24.9% compared to 31.1%, respectively).

**Table 2:** Descriptive statistics of the study sample overall and by language, N (%)

	**Total** ***N* = 4107**	**Runyoro** ***N* = 2206 (53.7)**	**Luganda** ***N* = 1901 (46.3)**
**Age, median (IQR)**	29 (21–39)	29 (22–41)	28 (20–38)
**Female**	2444 (59.5)	1276 (57.8)	1168 (61.4)
**Highest Education Level**			
No schooling	168 (4.1)	107 (4.9)	61 (3.2)
Primary	1883 (45.8)	1025 (46.5)	858 (45.1)
Secondary	1801 (43.9)	925 (41.9)	876 (46.1)
Tertiary	255 (6.2)	149 (6.8)	106 (5.6)
**Socioeconomic Status**			
Lowest	872 (21.3)	265 (12.0)	607 (32.1)
Lower	870 (21.3)	393 (17.8)	477 (25.2)
Higher	1042 (25.5)	599 (27.2)	443 (23.4)
Highest	1308 (32.0)	945 (42.9)	363 (19.2)
**Community Type**			
Urban	1419 (34.6)	797 (36.1)	622 (32.7)
Semi-urban	1277 (31.1)	549 (24.9)	728 (38.3)
Rural	1411 (34.4)	860 (39.0)	551 (29.0)
**PHQ-9**^**a**^			
Mean (SD), range 0–27	2.1 (2.4)	2.2 (2.3)	1.9 (2.4)
Median (IQR)	2 (0–3)	2 (0–3)	1 (0–3)

Percentages may not sum to 100 due to rounding. *IQR* interquartile range, *SD* standard deviations

^a^Depression symptoms measured with the Patient Health Questionnaire-9

The frequency of endorsing specific scale items were similar between Runyoro- and Luganda-speaking participants (Table 3); the most (i.e., item 1) and least (i.e., items 5 and 7) endorsed items were the same in both samples.

**Table 3:** GAD-7 item descriptives and factor loadings in Runyoro and Luganda

GAD Item	Runyoro (*N* = 2206)						Luganda (*N* = 1901)					
	**Item score**^**a**^	**% responses in each category**^**b**^				**λ**^**c**^	**Item score**^**a**^	**% responses in each category**^**b**^				**λ**^**c**^
		**0**	**1**	**2**	**3**			**0**	**1**	**2**	**3**	
**GAD1** “feeling nervous”	0.57 (0.60)	48.6	46.5	4.6	0.3	0.81	0.47 (0.64)	59.5	35.0	4.2	1.3	0.84
**GAD2** “unstoppable worrying”	0.52 (0.62)	54.1	39.6	5.9	0.3	0.92	0.35 (0.64)	72.6	21.1	4.8	1.5	0.96
**GAD3** “worrying too much”	0.30 (0.54)	74.0	22.3	3.4	0.3	0.78	0.22 (0.57)	84.1	10.5	4.2	1.2	0.92
**GAD4** “trouble relaxing”	0.14 (0.41)	87.9	10.0	1.9	0.1	0.67	0.12 (0.41)	90.5	7.5	1.4	0.6	0.80
**GAD5** “being restless”	0.04 (0.24)	96.7	2.4	0.8	-	0.75	0.06 (0.31)	95.9	2.7	0.9	0.4	0.76
**GAD6** “easily annoyed”	0.22 (0.45)	79.0	19.6	1.3	0.1	0.58	0.19 (0.47)	83.7	13.7	2.2	0.4	0.74
**GAD7** “feeling afraid”	0.13 (0.36)	88.3	10.9	0.9	-	0.67	0.06 (0.30)	95.0	4.0	0.7	0.3	0.68

λ = factor loading

^a^Item scores presented as mean (standard deviation)

^b^0 = Not at all, 1 = Several days, 2 = More than half the days, 3 = Nearly every day

^c^Item loadings for a one-factor structure using confirmatory factor analysis (CFA)

Assessing construct validity, the factor loadings in each scale were moderate to strong, ranging from 0.58 to 0.92 in Runyoro and from 0.68 to 0.96 in Luganda (Table 3). All factor loadings were higher in the Luganda-speaking sample compared to the Runyoro-speaking one. Model fit indices supported a one-factor structure of GAD-7 in both languages (Table 4), with an acceptable RMSEA value of 0.097 for both the Runyoro- and Luganda-translated GAD-7, and all CFI and TLI values above 0.96, indicating a good fit. Results of invariance testing by sex are provided as a Supplementary file.

**Table 4:** Psychometric properties of GAD-7 translated into Runyoro and Luganda

	**Runyoro** ***N* = 2206**	**Luganda** ***N* = 1901**
**1-Factor Confirmatory Factor Analysis**^**a**^		
Root Mean Square Error of Approximation (RMSEA)	0.097	0.097
Comparative Fit Index (CFI)	0.977	0.989
Tucker-Lewis Index (TLI)	0.966	0.983
**Internal Consistency**		
Revelle’s omega total	0.85	0.90
Cronbach’s alpha	0.77	0.84
**Concurrent Validity**^**b**^, r (*p*-value)		
PHQ-9	0.67 (*p* < 0.01)	0.71 (*p* < 0.01)

^a^RMSEA < 0.1 indicate acceptable, CFI and TLI > 0.9 indicate good model fit

^b^Estimated with Pearson’s correlation coefficient

The internal consistency coefficients indicated good (Revelle’s omega total = 0.85) and excellent (Revelle’s omega total = 0.90) internal consistency for the Runyoro- and Luganda-speaking sample, respectively (Table 4).

The correlation between GAD-7 and PHQ-9 was moderate in the Runyoro-speaking sample (*r* = 0.67, *p* < 0.01) and strong in the Luganda-speaking one (*r* = 0.71, *p* < 0.01), indicating that the translated anxiety scales had good concurrent validity with depression symptomology (Table 4).

GAD-7 median summed scores were low among both Runyoro- and Luganda-speaking participants (1 and 0, respectively; Table 5); 34.7% (765/2206) of Runyoro-speaking participants in Hoima and 50.1% (953/1901) of Luganda-speaking participants in Wakiso had a GAD-7 score of 0. When considering GAD-7 categorically, 91% of the Runyoro- and Luganda-speaking samples reported no or minimal anxiety symptoms. Using the dichotomous cut-off, 1.1% of the Runyoro-speaking sample screened positive for likely having an anxiety disorder (Table 2), with a higher prevalence among females compared to males (1.8% [23/1276] versus 0.2% [2/930], respectively). Similarly, 1.9% of the Luganda-speaking sample screened positive for likely having an anxiety disorder, with a higher prevalence among females compared to males (2.6% [30/1168] versus 1.0% [7/733], respectively).

**Table 5:** Descriptives statistics for GAD-7 overall and by language, N (%)

	**Total** ***N* = 4107**	**Runyoro** ***N* = 2206**	**Luganda** ***N* = 1901**	***p*-value**^**a**^
**Mean (SD), range 0–21**	1.8 (2.3)	1.9 (2.2)	1.5 (2.5)	< 0.001
**Median (IQR)**	1 (0–3)	1 (0–3)	0 (0–2)	< 0.001
**GAD-7 Categories, n (%)**^**b**^				0.007
No or minimal	3740 (91.1)	2004 (90.8)	1736 (91.3)	
Mild	305 (7.4)	177 (8.0)	128 (6.7)	
Moderate	51 (1.2)	24 (1.1)	27 (1.4)	
Severe	11 (0.3)	1 (0.0)	10 (0.5)	
**GAD-7 Dichotomized, n (%)**^**c**^				0.033
Anxiety disorder unlikely	4045 (98.5)	2181 (98.9)	1864 (98.1)	
Likely anxiety disorder	62 (1.5)	25 (1.1)	37 (1.9)	

*SD* standard deviations, *IQR* interquartile range

^a^Calculated using t-test (mean) or Wilcoxon rank-sum test (median) for continuous variables and Pearson's chi-squared test for categorical variables, comparing languages

^b^Anxiety categories: minimal 0–4, mild 5–9, moderate 10–14, severe 15–21

^c^Likely anxiety disorder: GAD-7 ≥ 10

## Discussion

Psychometrically, the Runyoro-translated GAD-7 had a one-factor structure, good internal consistency, and correlated moderately with depression symptomology as measured by the PHQ-9. The Luganda-translated GAD-7 also had a one-factor structure, excellent internal consistency, and correlated strongly with the PHQ-9. In both population-based samples, the prevalence of anxiety symptoms was low.

Both the Runyoro- and the Luganda-translated GAD-7 scales performed well in terms of the psychometric components that were assessed. Our findings are in line with previous research reporting good psychometric properties of the GAD-7 in different African settings. Studies among Ghanaian high school students [32], people living with HIV in Kenya [15], and primary care patients in Zimbabwe [33] confirmed its unidimensional factorial structure and good internal consistency. The studies in Ghana and Kenya also reported that the GAD-7 correlated with the PHQ-9, in line with our findings. In contrast, an evaluation among Sesotho-speaking South African tuberculosis patients found that a modified two-factor structure, comprising somatic and cognitive-emotional symptoms, fit their data better than a unidimensional model [34]. Invariance testing indicated that a one-factor model had suboptimal fit for Runyoro males, however these also had a very low estimated prevalence of anxiety and therefore we judge a one-factor structure to provide the best fit in both languages based on the more robust non-stratified analyses.

Comparing with population studies of anxiety disorders in other African countries, prevalence estimates in our sample were in the lower range of what has been previously reported [19]. A 2022 systematic review reported a sizeable increase in pooled anxiety prevalence in Africa during the COVID-19 pandemic to 47%, with estimates based on the GAD-7 ranging from 24 to 76% [35]. This review pooled studies using different screening tools and diagnostic instruments, complicating the interpretation of findings. However, our findings are comparable to studies from other East African nations. A 2013 community study in Western Kenya using the Clinical Interview Schedule-Revised found a 1% prevalence of GAD and 4% of any anxiety disorder [36]. In Rwanda, a nationally representative population survey in 2018 using the semi-diagnostic Mini International Neuropsychiatric Interview (MINI) found minimal levels of GAD (< 1%), but instead higher levels of panic disorder (8%), OCD (4%), and PTSD (4%) than estimates for neighbouring countries [37]. No general population studies from Tanzania reporting prevalences of anxiety disorders could be identified.

In the preliminary subgroup analysis by sex, a greater proportion of females had likely anxiety disorders compared to males, which supports worldwide findings regarding anxiety. However, the overall low prevalence contrasts with the estimated 20% prevalence of anxiety disorders in Uganda in the previously mentioned 2021 review [20]. Community studies not included in that review found adult GAD prevalences of 13% using the MINI in rural South-Western parts of the country in 2022 [38], and 11% in villages in Central Uganda using GAD-7 ≥ 10 as cut-off in 2020 [12]. Of note is that the latter study used a different Luganda version of GAD-7, locally adapted by researchers from Makerere University using a more sophisticated translation process. Low sensitivities and specificities of GAD-7 to detect anxiety disorders have been reported even when it was found to be psychometrically robust [39]. It is also important to consider that the prevalence of anxiety disorders in our sample may be even lower than the estimated 1.5%, considering the frequent overestimation of mental disorder prevalence by screening tools and assuming sensitivities and specificities of GAD-7 in the range of published results.

“Anxiety” can be understood as a process of creating an unpleasant feeling of tension, likely existing among all humans. However, the symptoms through which this tension is expressed and categorized into “anxiety disorders” were shaped by locally prevailing norms in European and US-American societies. Given the wide variation in estimates of anxiety symptoms/disorders globally and building on extensive transcultural research [40–43], one may question the appropriateness of attempting to measure anxiety uniformly worldwide. On the one hand, through the global spread of European and US-American psychiatric traditions and their conceptualizations of psychopathology, “anxiety” (like “depression”) became a de facto standard term and entered cultural spheres far from the term's origins [44]. On the other hand, a long tradition of anthropical research has been working side by side with the medical profession to try to understand the local aspects of the international terminology [45]. Our findings imply that anxiety disorders as conceptualized in Euro-American psychiatry and screened through GAD-7 are uncommon in this sample (i.e., GAD-7 is a robust measure but the real prevalence is low), or that individuals with more severe anxiety symptoms were systematically missed (i.e., GAD-7 is a robust measure and the real prevalence is higher than we found), or that local idioms are required to accurately measure the occurrence of anxiety (i.e., the real prevalence is higher than found and the current formulations of GAD-7 are inadequate). Prior qualitative research by the authors in the same population showed that community members experienced symptoms of mental distress, such as social withdrawal and “thinking too much”, and largely attributed these to contextual factors (such as economic insecurity). Discussion of mental distress on a social level was culturally taboo [46]. The discrepancy between this qualitative work and estimates of anxiety in the present study underscores a potential mismatch between conceptualizations of mental disorders in ICD and DSM and how “anxiety” may manifest and be understood in this setting. To strengthen future research and clinical practice, we recommend validating the Runyoro and Luganda GAD-7 against a diagnostic gold standard or, better yet, to develop more nuanced local measures of “anxiety”.

Substantial efforts have been made to put a stronger emphasis on culturally distinct conceptualizations when measuring human illness and wellness including the Shona Symptom Questionnaire [47], a Mozambican combined screening tool for depression and anxiety [48], and a South Sudanese assessment to capture local idioms of distress and mental illness [49]. In Uganda, a visual depression screening tool has been developed for low literacy settings [50]. These developments are promising examples of contextually adapted measures.

### Strengths and limitations

To our knowledge, this study is the first to examine certain psychometric properties of the GAD-7 translated into any Ugandan languages. Other strengths include the general population sample and large sample size, making this one of the largest investigations of anxiety symptoms in East Africa to date. Our samples cover a wide age range, large populations of both males and females, and diverse settings including semi-urban communities, which have received less in focus in mental health research than binary rural–urban categorizations which do not reflect actual urbanization processes.

Potential limitations include a potential selection bias towards individuals with minimal or mild anxiety symptoms. Those with moderate or severe symptoms may have avoided participation. Reasons for this could include logistical barriers, that participation would create additional anxiety, or that affected individuals avoid other community members due to stigma. The Runyoro and Luganda versions of GAD-7 were translated using a simplified process and considered to correspond in meaning by senior bilingual researchers, whose understanding of the items may not be consistent with that of the general population and the translations thus may lack salient local idioms of anxiety. Given only minor differences between our GAD-7 Luganda version and the one used by Makerere researchers, it is unlikely that different translations alone can explain the differing results. Modifications of the one-factor model for the Runyoro version might be considered in future work. We could not evaluate additional aspects of validity and reliability, such as convergent validity and test–retest reliability, because these data were not collected. Furthermore, our statistical approach did not address potential clustering within households as this was deemed of minor relevance. Using a screening tool which so far lacks clinically validated sensitivity and specificity in our settings, we were unable to calculate how different cut-off scores would impact the estimated population prevalence of likely anxiety disorders.

## Conclusions

GAD-7 was translated into Runyoro and Luganda, two Ugandan languages, and both translations showed good psychometric properties. The prevalence of likely anxiety disorders was low in this diverse large Ugandan population sample, the largest population study on anxiety in Uganda to date. The low prevalence could partly be due to individuals with more severe anxiety symptoms not participating or GAD-7 not including local idioms of anxiety in the questionnaire. Further clinical validation is required.

## Supplementary Information

Supplementary Material 1.

## Data Availability

The data that support the findings of this study are available from Africa Medical and Behavioural Sciences Organization but restrictions apply to the availability of these data, which were used under license for the current study, and so are not publicly available. Data are however available from the authors upon reasonable request and with permission of Africa Medical and Behavioural Sciences Organization.

## Abbreviations

AMBSO: Africa Medical and Behavioural Sciences Organization
APHS: AMBSO Population Health Surveillance
CEFR: Common European Framework of Reference for Languages
CFA: Confirmatory factor analysis
CFI: Comparative Fit Index
DSM: Diagnostic and Statistical Manual of Mental Disorders
GAD: Generalized anxiety disorder
GAD-7: Generalized Anxiety Disorder-7 scale
IQR: Interquartile range
OCD: Obsessive-compulsive disorder
PHQ-9: Patient Health Questionnaire-9
PTSD: Post-traumatic stress disorder
RMSEA: Root Mean Square Error of Approximation
SD: Standard deviations
TLI: Tucker-Lewis Index

## References

[1] GBD 2019 Mental Disorders Collaborators. Global, regional, and national burden of 12 mental disorders in 204 countries and territories, 1990–2019: a systematic analysis for the Global Burden of Disease Study 2019. Lancet Psychiatry. 2022;9(2):137–50. 35026139 10.1016/S2215-0366(21)00395-3 PMC8776563

[2] Patel V, Saxena S, Lund C, Thornicroft G, Baingana F, Bolton P, et al. The Lancet Commission on global mental health and sustainable development. Lancet. 2018;392(10157):1553–98. 30314863 10.1016/S0140-6736(18)31612-X

[3] Mostert CM, Nesic O, Udeh-Momoh C, Khan M, Thesen T, Bosire E, et al. Who should pay the bill for the mental health crisis in Africa? Public Health Pract. 2024;1(7):100458. 10.1016/j.puhip.2023.100458 PMC10770737 38187932

[4] Spitzer RL, Kroenke K, Williams JBW, Löwe B. A Brief Measure for Assessing Generalized Anxiety Disorder: The GAD-7. Arch Intern Med. 2006;166(10):1092–7. 16717171 10.1001/archinte.166.10.1092

[5] Kroenke K, Spitzer RL, Williams JBW. The PHQ-9. J Gen Intern Med. 2001;16(9):606–13. 11556941 10.1046/j.1525-1497.2001.016009606.x PMC1495268

[6] Kroenke K, Spitzer RL, Williams JBW, Löwe B. The Patient Health Questionnaire Somatic, Anxiety, and Depressive Symptom Scales: a systematic review. Gen Hosp Psychiatry. 2010;32(4):345–59. 20633738 10.1016/j.genhosppsych.2010.03.006

[7] Screener Overview. Patient Health Questionnaire (PHQ) Screeners. https://www.phqscreeners.com/select-screener/. Accessed 10 Oct 2024.

[8] Hinton DE, Lewis-Fernández R. “Idioms of distress” (culturally salient indicators of distress) and anxiety disorders. In: Schneier F, Simpson HB, Lewis-Fernández R, Neria Y, editors. Anxiety Disorders: Theory, Research and Clinical Perspectives. Cambridge: Cambridge University Press; 2010. p. 127–38.

[9] Flaherty JA, Gaviria FM, Pathak D, Mitchell T, Wintrob R, Richman JA, et al. Developing instruments for cross-cultural psychiatric research. J Nerv Ment Dis. 1988;176(5):257–63. 3367140

[10] Prince MJ. Cross-Cultural Research Methods and Practice. In: Patel V, Minas H, Cohen A, Prince M, editors. Global Mental Health: Principles and Practice. Oxford University Press; 2013. p. 63–81.

[11] Mugisha J, Byansi PK, Kinyanda E, Bbosa RS, Van Damme T, Vancampfort D. Moderate to severe generalized anxiety disorder symptoms are associated with physical inactivity in people with HIV/AIDS: a study from Uganda. Int J STD AIDS. 2021;32(2):170–5. 33323069 10.1177/0956462420942992

[12] Akena D, Kiguba R, Muwhezi WW, Kwesiga B, Kigozi G, Lukwata H, et al. The prevalence and factors associated with mental disorders in a community setting in central Uganda. PLoS ONE. 2023;18(5):e0285091. 37141327 10.1371/journal.pone.0285091 PMC10159349

[13] Sidik SM, Arroll B, Goodyear-Smith F. Validation of the GAD-7 (Malay version) among women attending a primary care clinic in Malaysia. J Prim Health Care. 2012;4(1):5–11. 22377544

[14] Villarreal-Zegarra D, Paredes-Angeles R, Mayo-Puchoc N, Arenas-Minaya E, Huarcaya-Victoria J, Copez-Lonzoy A. Psychometric properties of the GAD-7 (General Anxiety Disorder-7): a cross-sectional study of the Peruvian general population. BMC Psychol. 2024;12(1):183. 38566138 10.1186/s40359-024-01688-8 PMC10985987

[15] Nyongesa MK, Mwangi P, Koot HM, Cuijpers P, Newton CRJC, Abubakar A. The reliability, validity and factorial structure of the Swahili version of the 7-item generalized anxiety disorder scale (GAD-7) among adults living with HIV from Kilifi, Kenya. Ann Gen Psychiatry. 2020;28(19):62. 10.1186/s12991-020-00312-4 PMC7594456 33133222

[16] Levis B, Benedetti A, Ioannidis JPA, Sun Y, Negeri Z, He C, et al. Patient Health Questionnaire-9 scores do not accurately estimate depression prevalence: individual participant data meta-analysis. J Clin Epidemiol. 2020;1(122):115-128.e1. 10.1016/j.jclinepi.2020.02.002 32105798

[17] Mughal AY, Devadas J, Ardman E, Levis B, Go VF, Gaynes BN. A systematic review of validated screening tools for anxiety disorders and PTSD in low to middle income countries. BMC Psychiatry. 2020;20(1):338. 32605551 10.1186/s12888-020-02753-3 PMC7325104

[18] Plummer F, Manea L, Trepel D, McMillan D. Screening for anxiety disorders with the GAD-7 and GAD-2: a systematic review and diagnostic metaanalysis. Gen Hosp Psychiatry. 2016;1(39):24–31. 10.1016/j.genhosppsych.2015.11.005 26719105

[19] Greene MC, Yangchen T, Lehner T, Sullivan PF, Pato CN, McIntosh A, et al. The epidemiology of psychiatric disorders in Africa: a scoping review. Lancet Psychiatry. 2021;8(8):717–31. 34115983 10.1016/S2215-0366(21)00009-2 PMC9113063

[20] Opio JN, Munn Z, Aromataris E. Prevalence of Mental Disorders in Uganda: a Systematic Review and Meta-Analysis. Psychiatr Q. 2022;93(1):199–226. 34427855 10.1007/s11126-021-09941-8

[21] Mugamba S, Ziegel L, Bulamba RM, Kyasanku E, Johansson Århem K, Sjöland CF, et al. Cohort Profile: The Africa Medical and Behavioral Sciences Organization (AMBSO) Population Health Surveillance (APHS) in rural, semi-urban and urban Uganda. Int J Epidemiol. 2023;52(2):e116–24. 35962975 10.1093/ije/dyac164 PMC10114126

[22] Wealth-Index-Construction. The DHS Program. https://dhsprogram.com/topics/wealth-index/Wealth-Index-Construction.cfm. Accessed 10 Oct 2024.

[23] Miller AP, Espinosa da Silva C, Ziegel L, Mugamba S, Kyasanku E, Bulamba RM, et al. Construct validity and internal consistency of the Patient Health Questionnaire-9 (PHQ-9) depression screening measure translated into two Ugandan languages. Psychiatry Res Commun. 2021;1(2):100002. 35187539 10.1016/j.psycom.2021.100002 PMC8855962

[24] Rosseel Y. lavaan: An R Package for Structural Equation Modeling. J Stat Softw. 2012;24(48):1–36.

[25] Floyd FJ, Widaman KF. Factor analysis in the development and refinement of clinical assessment instruments. Psychol Assess. 1995;7(3):286–99.

[26] Kenny DA, Kaniskan B, McCoach DB. The Performance of RMSEA in Models With Small Degrees of Freedom. Sociol Methods Res. 2015;44(3):486–507.

[27] Streiner DL. Starting at the Beginning: An Introduction to Coefficient Alpha and Internal Consistency. J Pers Assess. 2003;80(1):99–103. 12584072 10.1207/S15327752JPA8001_18

[28] McNeish D. Thanks coefficient alpha, we’ll take it from here. Psychol Methods. 2018;23(3):412–33. 28557467 10.1037/met0000144

[29] Revelle W. psych: Procedures for Personality and Psychological Research. 2017. https://CRAN.R-project.org/package=psych. Accessed 10 Oct 2024.

[30] Song J, Howe E, Oltmanns JR, Fisher AJ. Examining the Concurrent and Predictive Validity of Single Items in Ecological Momentary Assessments. Assessment. 2023;30(5):1662–71. 36004406 10.1177/10731911221113563 PMC10248304

[31] Akoglu H. User’s guide to correlation coefficients. Turk J Emerg Med. 2018;18(3):91–3. 30191186 10.1016/j.tjem.2018.08.001 PMC6107969

[32] Adjorlolo S. Generalised anxiety disorder in adolescents in Ghana: Examination of the psychometric properties of the Generalised Anxiety Disorder-7 scale. Afr J Psychol Assess. 2019;1:7.

[33] Chibanda D, Verhey R, Gibson LJ, Munetsi E, Machando D, Rusakaniko S, et al. Validation of screening tools for depression and anxiety disorders in a primary care population with high HIV prevalence in Zimbabwe. J Affect Disord. 2016;1(198):50–5. 10.1016/j.jad.2016.03.006 27011359

[34] Kigozi G. Construct validity and reliability of the generalised anxiety disorder-7 scale in a sample of tuberculosis patients in the Free State Province, South Africa. South Afr J Infect Dis. 2021;36(1):6. 10.4102/sajid.v36i1.298 PMC8424773 34522696

[35] Bello UM, Kannan P, Chutiyami M, Salihu D, Cheong AMY, Miller T, et al. Prevalence of Anxiety and Depression Among the General Population in Africa During the COVID-19 Pandemic: A Systematic Review and Meta-Analysis. Front Public Health. 2022;10:814981. 35655463 10.3389/fpubh.2022.814981 PMC9152218

[36] Jenkins R, Othieno C, Ongeri L, Sifuna P, Ongecha M, Kingora J, et al. Common mental disorder in Nyanza province, Kenya in 2013 and its associated risk factors –an assessment of change since 2004, using a repeat household survey in a demographic surveillance site. BMC Psychiatry. 2015;15(1):309. 26651332 10.1186/s12888-015-0693-5 PMC4673710

[37] Kayiteshonga Y, Sezibera V, Mugabo L, Iyamuremye JD. Prevalence of mental disorders, associated co-morbidities, health care knowledge and service utilization in Rwanda – towards a blueprint for promoting mental health care services in low- and middle-income countries? BMC Public Health. 2022;22(1):1858. 36199102 10.1186/s12889-022-14165-x PMC9533613

[38] Kalungi A, Kinyanda E, Akena DH, Gelaye B, Ssembajjwe W, Mpango RS, et al. Prevalence and correlates of common mental disorders among participants of the Uganda Genome Resource: Opportunities for psychiatric genetics research. Mol Psychiatry. 2024;14:1–9. 10.1038/s41380-024-02665-8 PMC11649557 39003415

[39] Sawaya H, Atoui M, Hamadeh A, Zeinoun P, Nahas Z. Adaptation and initial validation of the Patient Health Questionnaire – 9 (PHQ-9) and the Generalized Anxiety Disorder – 7 Questionnaire (GAD-7) in an Arabic speaking Lebanese psychiatric outpatient sample. Psychiatry Res. 2016;30(239):245–52. 10.1016/j.psychres.2016.03.030 27031595

[40] Hofmann SG, Hinton DE. Cross-Cultural Aspects of Anxiety Disorders. Curr Psychiatry Rep. 2014;16(6):450. 24744049 10.1007/s11920-014-0450-3 PMC4037698

[41] Kaiser BN, Jo WL. Culture-bound syndromes, idioms of distress, and cultural concepts of distress: New directions for an old concept in psychological anthropology. Transcult Psychiatry. 2019;56(4):589–98. 31347475 10.1177/1363461519862708 PMC6724704

[42] Patel V, Gwanzura F, Simunyu E, Lloyd K, Mann A. The phenomenology and explanatory models of common mental disorder: a study in primary care in Harare. Zimbabwe Psychol Med. 1995;25(6):1191–9. 8637949 10.1017/s003329170003316x

[43] Wasil AR, Venturo-Conerly KE, Gillespie S, Osborn TL, Weisz JR. In Their Own Words: Using Open-Ended Assessment to Identify Culturally Relevant Concerns among Kenyan Adolescents. Cult Med Psychiatry. 2022;46(2):297–321. 33528725 10.1007/s11013-020-09706-1

[44] Watters E. Crazy like us: The globalization of the American psyche. New York, NY, US: Free Press; vii, 306 p. Crazy like us: The globalization of the American psyche; 2010.

[45] Kaiser BN, Haroz EE, Kohrt BA, Bolton PA, Bass JK, Hinton DE. “Thinking too much”: A systematic review of a common idiom of distress. Soc Sci Med. 2015;1(147):170–83. 10.1016/j.socscimed.2015.10.044 PMC4689615 26584235

[46] Miller AP, Ziegel L, Mugamba S, Kyasanku E, Wagman JA, Nkwanzi-Lubega V, et al. Not Enough Money and Too Many Thoughts: Exploring Perceptions of Mental Health in Two Ugandan Districts Through the Mental Health Literacy Framework. Qual Health Res. 2021;31(5):967–82. 33451275 10.1177/1049732320986164 PMC8628861

[47] Patel V, Simunyu E, Gwanzura F, Lewis G, Mann A. The Shona Symptom Questionnaire: the development of an indigenous measure of common mental disorders in Harare. Acta Psychiatr Scand. 1997;95(6):469–75. 9242841 10.1111/j.1600-0447.1997.tb10134.x

[48] Belus JM, Muanido A, Cumbe VFJ, Manaca MN, Wagenaar BH. Psychometric Validation of a Combined Assessment for Anxiety and Depression in Primary Care in Mozambique (CAD-MZ). Assessment. 2022;29(8):1890–900. 34353141 10.1177/10731911211032285 PMC8816971

[49] Ng LC, Solomon JS, Ameresekere M, Bass J, Henderson DC, Chakravarty S. Development of the South Sudan Mental Health Assessment Scale. Transcult Psychiatry. 2022;59(3):274–91. 34898333 10.1177/13634615211059711 PMC9575559

[50] Akena D, Joska J, Musisi S, Stein DJ. Sensitivity and Specificity of a Visual Depression Screening Instrument Among HIV-Positive Individuals in Uganda, an Area with Low Literacy. AIDS Behav. 2012;16(8):2399–406. 22810893 10.1007/s10461-012-0267-1

