# Supplementary material for: Prevalence of anxiety symptoms in a Ugandan population sample and psychometric properties of the Generalized Anxiety Disorder-7 scale (GAD-7) in Luganda and Runyoro
Source: BMC Psychiatry. 2025 May 19;25:502. doi: 10.1186/s12888-025-06944-8 (PMC12090614; doi:10.1186/s12888-025-06944-8)
Supplement: Supplementary file 1 — Supplementary Material 1. [file 12888_2025_6944_MOESM1_ESM.docx]

Invariance testing results for a one-factor structure of GAD-7 in Luganda and Runyoro, separate for males/females.

|  | Luganda  Female | Luganda  Male | Runyoro Female | Runyoro  Male |
| --- | --- | --- | --- | --- |
| RMSEA | 0.070 | 0.093 | 0.072 | 0.185 |
| CFI | 0.994 | 0.989 | 0.992 | 0.900 |
| TLI | 0.991 | 0.983 | 0.988 | 0.850 |
